# Assessment of bone response to systemic therapy in an EORTC trial: preliminary experience with the use of collagen cross-link excretion

**DOI:** 10.1038/sj.bjc.6690506

**Published:** 1999-04

**Authors:** J Vinholes, R Coleman, D Lacombe, C Rose, M Tubiana-Hulin, P Bastit, J Wildiers, J Michel, R Leonard, J Nortier, F Mignolet, J Ford

**Affiliations:** EORTC (European Organization for Research and Treatment of Cancer), Breast Cancer Cooperative Group, Brussels, Belgium; EORTC Data Center, Brussels, Belgium; Novartis, Basel, Switzerland

**Keywords:** bone metastases, assessment of response, breast cancer, bone resorption markers, tumour markers

## Abstract

This study was designed to evaluate new bone resorption and tumour markers as possible alternatives to serial plain radiographs for the assessment of response to treatment. Thirty-seven patients with newly diagnosed bone metastases from breast cancer, randomized to receive oral pamidronate or placebo tablets in addition to anticancer treatment within the context of a multicentre EORTC trial, who were both assessable for radiographic response in bone and had serum and urine samples collected for more than 1 month were studied. The markers of bone metabolism measured included urinary calcium (uCa), hydroxyproline (hyp), the N-telopeptide cross-links of type I collagen (NTx) and total alkaline phosphatase. The tumour markers measured were CA15-3 and cancer-associated serum antigen (CASA). Before treatment, levels of Ntx, uCa and Hyp were elevated in 41%, 24% and 28% respectively, and CA15-3 and CASA increased in 69% and 50%. For assessment of response and identification of progression, Ntx was the most useful bone marker. All markers behaved similarly in no change (NC) and partial response (PR) patients. There was a significant difference (*P* ≤ 0.05) in Ntx levels (compared to baseline) at 1 and 4 months and in CA15-3/CASA at 4 months between patients with PR or NC and those with progressive disease (PD), and at 4 months between those with time to progression (TP) > 7 and those with TP ≤ 7 months. The diagnostic efficiency (DE) for prediction of PD following a > 50% increase in Ntx or CA15-3 was 78% and 62% respectively. An algorithm to predict response to therapy has been developed for future prospective evaluation. © 1999 Cancer Research Campaign


*British Journal of Cancer* (1999) **80**(1/2), 221–228
© 1999 Cancer Research Campaign
Article no. bjoc.1998.0343

### Assessment of bone response to systemic therapy in an EORTC trial: preliminary experience with the use of collagen cross-link excretion

**J Vinholes[1], R Coleman[1], D Lacombe[2], C Rose[1], M Tubiana-Hulin[1], P Bastit[1], J Wildiers[1], J Michel[1], R Leonard[1], J Nortier[1], F Mignolet[2] and J Ford[3]**

[1]EORTC (European Organization for Research and Treatment of Cancer), Breast Cancer Cooperative Group, Brussels, Belgium; [2]EORTC Data Center, Brussels, Belgium; [3]Novartis, Basel, Switzerland

**Summary** This study was designed to evaluate new bone resorption and tumour markers as possible alternatives to serial plain radiographs for the assessment of response to treatment. Thirty-seven patients with newly diagnosed bone metastases from breast cancer, randomized to receive oral pamidronate or placebo tablets in addition to anticancer treatment within the context of a multicentre EORTC trial, who were both assessable for radiographic response in bone and had serum and urine samples collected for more than 1 month were studied. The markers of bone metabolism measured included urinary calcium (uCa), hydroxyproline (hyp), the N-telopeptide cross-links of type I collagen (NTx) and total alkaline phosphatase. The tumour markers measured were CA15-3 and cancer-associated serum antigen (CASA). Before treatment, levels of Ntx, uCa and Hyp were elevated in 41%, 24% and 28% respectively, and CA15-3 and CASA increased in 69% and 50%. For assessment of response and identification of progression, Ntx was the most useful bone marker. All markers behaved similarly in no change (NC) and partial response (PR) patients. There was a significant difference ($P \leq 0.05$) in Ntx levels (compared to baseline) at 1 and 4 months and in CA15-3/CASA at 4 months between patients with PR or NC and those with progressive disease (PD), and at 4 months between those with time to progression (TP) > 7 and those with TP ≤ 7 months. The diagnostic efficiency (DE) for prediction of PD following a > 50% increase in Ntx or CA15-3 was 78% and 62% respectively. An algorithm to predict response to therapy has been developed for future prospective evaluation.

**Keywords:** bone metastases; assessment of response; breast cancer; bone resorption markers; tumour markers

A variety of treatments, including radiotherapy, endocrine treatment, chemotherapy and bisphosphonates, are used for the treatment of metastatic bone disease and evaluation of their effects is important for both routine clinical practice and research. The current methods used to assess response to these treatments are qualitative and routinely include plain radiographs, radionuclide bone scans and, in particular situations, computerized tomography (CT).

The response assessment of bone metastases to therapy is notoriously difficult, as the events in the healing process are slow to evolve and quite subtle, with sclerosis of lytic lesions only beginning to appear 3–6 months after the start of therapy (Coleman, 1998). Confounding factors include the appearance of sclerosis in a previously normal area on the radiograph; this may indicate a new metastasis but equally may indicate a response, representing an example of the healing flare phenomenon within a lesion which was present at the start, but not destructive enough to be radiologically visible (Galasko, 1994). The evaluation of response in osteosclerotic lesions is even more difficult with most patients with sclerotic metastases eventually classified as either 'no change' (NC) to therapy or as unassessable; for these patients, decisions about the efficacy of treatment are based on symptomatic response or (when present) change in extraskeletal disease.

All these pitfalls in radiological assessment of response are reflected in the correlation of radiological response and the clinical course of bone metastases. Complete (CR) and partial responses (PR) in bone appear to be less frequent than in other tissues (Coleman and Rubens, 1985), and the survival prospects for patients with PR or NC are similar (Howell et al, 1988). In view of the limitations of the current imaging methods, patients with metastatic disease confined to the bone are frequently excluded from many therapeutic trials and patients with widespread metastatic disease (including bone) have changes in soft tissue or visceral disease as their main response parameters.

While recognizing that the changes seen on serial radiographs remain the 'gold standard' for evaluating response to therapy, alternative methods are urgently needed. Biochemical markers of bone turnover have shown some promise in the assessment of response in bone to systemic anticancer therapy (Coleman et al, 1988), as have tumour marker measurements (Robertson et al, 1991). Additionally, new assays have recently been developed to measure the cross-linking molecule at either the N-terminal part (NTx) (Hanson, 1992) or the C-terminal (Bonde et al, 1995) part (Crosslaps) of type I collagen, providing a simple, specific indication of the rate of bone resorption. We have previously shown that these markers are significantly increased in patients with bone metastases and that urine levels decrease in response to bisphosphonate treatment (Vinholes et al, 1996, 1997*a*, 1997*b*).

*Received 9 January 1998*
*Accepted 22 October 1998*

*Correspondence to:* RE Coleman, Yorkshire Cancer Research Department of Clinical Oncology, Weston Park Hospital, Sheffield S10 2SJ, UK

The aims of this study were to evaluate biochemical resorption markers in breast cancer patients with bone metastases, and to correlate the biochemical changes with clinical and tumour marker response to treatment within a multicentre randomized trial. The study was conducted in the context of an EORTC (European Organization for Research and Treatment of Cancer) trial (protocol 10924) designed to assess the effect of oral pamidronate, in comparison to placebo, in reducing the rate of skeletal-related events. The trial was stopped after 18 months because other trials evaluating oral pamidronate were negative (unpublished data). In addition, there were concerns about its absorption and gastro-intestinal toxicity. These results will be reported elsewhere.

### PATIENTS AND METHODS

Ninety patients with histologically proven breast cancer and radiologically confirmed newly diagnosed bone metastases identified within the previous 6 weeks were randomized to receive oral pamidronate 300 mg daily or identical placebo tablets in addition to standard anticancer treatment as part of an EORTC multicentre, randomized, double-blind trial. The choice of specific anticancer treatment was left at the discretion of the patient's treating physician and included radiotherapy, chemotherapy and endocrine therapy.

Bone metastases were identified by radionuclide bone scan and confirmed by plain radiographs. In patients with a solitary bone metastasis, confirmation of bone involvement by CT or magnetic resonance imaging (MRI) scan was necessary. Involvement of other metastatic sites was allowed. No treatment with bisphosphonates within 6 months of randomization, or exposure to drugs affecting bone metabolism, was allowed except for corticosteroids taken for < 3 weeks duration. Other inclusion criteria included age > 18 years; performance status 0–2; serum calcium < 2.8 mmol $l^{-1}$ and life expectancy > 3 months. All patients gave written informed consent.

Eight of the centres contributing patients to the trial took part in this subprotocol exploring the use of alternative biochemical markers of response. Thirty-seven patients with serial plain radiographs, urine collections and serum samples were considered evaluable for both radiographic and biochemical response and are the subject of this report. However, one patient did not have serum collected for tumour markers.

### Response criteria

Radiographs of involved sites were performed at baseline, 4 months and at 3-monthly intervals thereafter. They were also performed each time there was either a change in systemic therapy or a skeletal event (fracture, radiotherapy to bone, hypercalcaemia, radiological progression or spinal cord compression).

For this report, the effects of different treatments on both bone and tumour markers were only evaluated for the first 7 months on treatment as very few patients had samples collected beyond this time. Only the response to first-line systemic treatment within this trial is reported, as only a few patients were assessable for response to subsequent treatments. Patients were also classified according to the time to progression (TP) into two groups (TP of ≥ 7 months and TP < 7 months).

Radiological response to treatment was evaluated according to the UICC criteria (Hayward et al, 1977). All radiographs were reviewed by two research fellows (J Vinholes and D Lacombe) in conjunction with the investigators and local radiologists, and without knowledge of the biochemical markers levels.

Subjective response to treatment was evaluated according to a pain score, as previously described (Coleman, 1994). The pain score was expressed as the percentage change from baseline. Clinical response to treatment was defined as ≥ 20% decrease in the pain score compared to baseline reported on at least two consecutive measurements. Patients with a decrease in pain of < 20% were considered clinical non-responders.

### Biochemical analysis

A fasting morning serum sample and a second voided urine sample were collected from patients at baseline, 1 month after trial entry and at 3-monthly intervals thereafter. Samples were also collected in the event of a change in systemic therapy or skeletal event. Serum measurements included full blood count, serum calcium (sCa) corrected for albumin, total alkaline phosphatase (tAP) creatinine and phosphate ($PO_4$). Bone resorption markers included urinary calcium (uCa), urinary hydroxyproline (hyp) and urinary N-telopeptide peptide-bound cross-links (NTx) excretion. Tumour markers measured included CA15-3 and CASA (cancer-associated serum antigen). All bone resorption and tumour markers were measured in batches in Sheffield. Samples were stored at < –20°C and shipped on dry ice.

NTx was measured by an enzyme-linked immunoabsorbent assay (ELISA) which uses a horseradish peroxidase labelled monoclonal antibody that recognizes an epitope in the N-telopeptide cross-linking domain of type I collagen (Osteomark™, Ostex®, Inc, Seattle, WA, USA). The detection limit was 20 nmol BCE (bone collagen equivalent). The intra-assay and inter-assay variability were 8% and 11% respectively. uCa was measured by a colorimetric assay, and hyp by a colorimetric assay with dimethylaminobenzaldehyde after acid hydrolysis and chloramine T oxidation. All the urinary markers were expressed as a ratio to creatinine excretion in the urine, measured using an automated chemistry analyser by the kinetic Jaffe method.

To establish a control group for the bone resorption markers, we had previously enrolled 40 healthy volunteers who were not taking any medication with affects on bone metabolism (Vinholes et al, 1997*a*).

### Statistical methods

As all markers showed a skewed distribution, data were logarithmically transformed and expressed as the geometric mean ± 2 standard deviation (s.d.). The baseline marker values of patients were expressed as the geometric mean with 95% confidence intervals. To compare patients with controls, we used the unpaired *t*-test after log transformation of the data and considered $P < 0.05$ as significant.

The response of each marker to treatment was expressed as the percentage change from baseline and, as these data were also not normally distributed, they were expressed as the geometric mean ± standard error of the mean. The paired *t*-test with Bonferroni correction was used to compare the percentage change for marker values at each time-point with the baseline value. For determining both the correlation between markers at baseline and after therapy, Pearson's correlation method was used. To compare changes in pain within and between the groups, we used the Wilcoxon signed-ranks test and the Mann–Whitney test respectively.

 © Cancer Research Campaign 1999

**Table 1** Scores assigned to changes in biochemical markers, tumour markers and pain response for use in an algorithm to predict objective radiological response to treatment

| | –1 | 0 | +1 |
|---|---|---|---|
| NTx | ≥ 30% fall | < 30% fall or < 30 rise | ≥ 30% rise |
| CA15-3 | ≥ 10% fall | < 10% fall or < 10% rise | ≥ 10% rise |
| tAP | ≥ 10% fall | < 10% fall or < 10% rise | ≥ 10% rise |
| Pain score | ≥ 20% fall | < 20% fall and no rise | > 0% rise |

NTx = N-telopeptide cross-links; tAP = total alkaline phosphatase.

The predictive value of changes in bone and tumour markers for identifying the response demonstrated by serial plain radiographs was calculated. The positive predictive value is the proportion of patients with positive test results who are correctly diagnosed radiologically. The negative predictive value is the proportion of patients with negative test results who are correctly diagnosed radiologically. Diagnostic efficiency (DE) is the number of patients with the correct diagnosis (true positive plus true negative) divided by the total number of patients. We calculated the predictive values and DE of a decrease in bone turnover markers and tumour markers of 10%, 20%, 30%, 40% and 50% from baseline at 1 month to predict for a partial response and/or no change at 4 months. We also calculated the predictive values and diagnostic efficiency of an increase in markers of 10%, 20%, 30%, 40% and 50% at 1 month to predict for progression at 4 months.

In an attempt to produce an algorithm to predict response, a preliminary analysis of changes in various possible combinations of markers and clinical response was performed. The cut-off value for an individual marker was based on the least clinically significant change – for Ntx this was 30% reduction (Blumsohn et al, 1994), 10% for both CA15-3 (Robertson et al, 1991) and alkaline phosphatase (Coleman et al, 1988) and 20% for the pain score (Vinholes et al, 1996, 1997*b*) – and the highest DE observed for each parameter. These cut-off values are summarized in Table 1. In each patient, the score for each variable was combined. Progressive disease was considered as any positive value obtained, and NC/PR as any negative value obtained or zero. These combinations were then compared with the radiological response and time to progression to determine whether they were of any predictive value.

**Table 2** Baseline characteristics of patients

| | Pamidronate (*n* = 17) | Placebo (*n* = 20) |
|---|---|---|
| Median age (range) | 60 (35–76) | 48 (38–70) |
| Post-menopausal | 11 | 12 |
| Bone only disease | 14 | 16 |
| Multiple bone metastases | 14 | 17 |
| Median time from diagnosis (months) | 24 | 24 |
| Previous adjuvant therapy | 9 | 15 |
| Systemic treatment | | |
| Chemotherapy | 3 | 10 |
| Endocrine therapy | 14 | 10 |
| Median pain score (range) | 3 (0–7) | 5.5 (0–9) |

### RESULTS

The baseline characteristics of the patients are shown in Table 2. No significant differences were observed between these groups, except for the type of anticancer treatment received. Fourteen patients in the pamidronate arm received hormone therapy (seven tamoxifen, four megestrol acetate, two aminogluthetimide and one goserelin) and only three chemotherapy (anthracycline-based regimen). In the placebo arm, ten received endocrine treatment (six tamoxifen, three megestrol acetate and one ovarian ablation) and ten chemotherapy (eight anthracycline-based regimens, one methotrexate-based regimen and one vinorelbine).

### Pre-treatment markers

Baseline values of the markers are shown in Table 3, except for tAP which, because it was measured at each centre by various methods, could not be reliably compared to our control patients. Patients in both groups had similar levels and are reported together. There was no difference in the levels of baseline markers with regard to menopausal status or between patients on chemotherapy or endocrine treatment.

The mean values of NTx and hyp were 50% and 25% higher, respectively, in patients than in controls, while uCa was lower in patients than in controls. CA15-3 and CASA were 36% and 11% higher in patients than in controls respectively. In these patients

**Table 3** Baseline bone resorption and tumour markers

| Marker | Units | Controls | Patients | Patients with increased value (%) |
|---|---|---|---|---|
| NTx | nmol BCE $mmol^{-1}$ | 55 (25–120) | 84 (66–107) | 41 |
| Hyp | mmol $mmol^{-1}$ | 24 (11–48) | 30 (26–35) | 24 |
| uCa | mmol $mmol^{-1}$ | 0.33 (0.13–0.53) | 0.27 (0.21–0.34) | 28 |
| CA15-3 | U $ml^{-1}$ | 35* | 48 (32–71) | 69 |
| CASA | U $ml^{-1}$ | 6[a] | 6.7 (4.4–10.1) | 50 |

[a]Upper limit of reference range. NTx = N-telopeptide cross-links; Hyp = hydroxyproline; uCa = urinary calcium; CASA = cancer-associated serum antigen.

© Cancer Research Campaign 1999

**Table 4** Correlations (*r* values) between bone resorption markers and tumour markers at baseline

| Marker | Ntx | Hyp | uCa | TAP | CA15-3 | CASA |
|---|---|---|---|---|---|---|
| NTx | 1 | | | | | |
| Hyp | 0.66[a] | 1 | | | | |
| uCa | 0.45[b] | 0.59[b] | 1 | | | |
| tAP | 0.57[b] | 0.53[b] | 0.37 | 1 | | |
| CA15-3 | 0.32 | 0.26 | 0.19 | 0.22 | 1 | |
| CASA | 0.23 | 0.19 | 0.13 | 0.12 | 0.52[b] | 1 |

[a]$P \leq 0.01$, [b] $P \leq 0.05$. NTx = N-telepeptide cross-links; Hyp = hydroxyproline; uCa = urinary calcium; CASA = cancer-associated serum antigen.

**Table 5** Predictive values of changes in markers at 1 month for response to treatment as determined at 4 months

| Marker (s) | PV + (%) | PV– (%) | DE (%) |
|---|---|---|---|
| NTx (≥ 30% fall) | 95 | 41 | 69 |
| CA15-3 (≥ 10% fall) | 82 | 41 | 61 |
| tAP (≥ 10% fall) | 94 | 47 | 69 |
| Pain score (≥ 20% fall) | 94 | 47 | 69 |
| NTx + CA15-3 | 95 | 35 | 44 |
| NTx + Pain score | 95 | 53 | 56 |
| tAP + Pain score | 89 | 53 | 53 |
| NTx + tAP | 95 | 58 | 64 |
| NTx + tAP + Pain score | 95 | 70 | 72 |
| NTx + CA15-3 + Pain score | 95 | 53 | 58 |
| NTx + tAP + CA15-3 | 100 | 53 | 56 |
| NTx + tAP + CA15-3 + Pain score | 100 | 58 | 61 |

PV + = positive predictive value; PV – = negative predictive value; DE = diagnostic efficiency. NTx = N-telopeptide cross-links; tAP = total alkaline phosphatase.

**Table 6** Predictive value for PD of a 50% increase in markers or pain whenever it occurs

| | PV+ (%) | PV– (%) | DE (%) |
|---|---|---|---|
| NTx | 81 | 76 | 78 |
| Hyp | 57 | 62 | 59 |
| uCa | 62 | 25 | 46 |
| tAP | 94 | 33 | 59 |
| CA15-3 | 75 | 52 | 62 |
| CASA | 50 | 52 | 51 |
| Pain score | 100 | 6 | 42 |

PV+ = positive predictive value; PV– = negative predictive value; DE = diagnostic efficiency. NTx = N-telopeptide cross-links; Hyp = hydroxyproline; uCa = urinary calcium; tAP = total alkaline phosphatase; CASA = cancer-associated serum antigen.

with early metastatic bone disease, tumour marker levels were more likely to be elevated than the bone resorption markers.

CA15-3 and CASA levels correlated with one another but not with the bone resorption markers, even when patients with sites of disease other than bone were excluded from the correlation. NTx correlated with hyp, but only partially with uCa. tAP correlated with NTx and hyp (Table 4).

### Assessment of response at 4 months

There was a significant difference in mean NTx levels between progressive disease (PD) patients versus NC or PR patients at 1 ($P \leq 0.05$) and 4 months ($P \leq 0.01$). Hydroxyproline only showed a difference between these groups at 4 months, while uCa could not differentiate between them (Figure 1). There was also a significant difference in the CA15-3 levels between PD patients versus NC or PR patients at 4 months ($P \leq 0.05$), but not at 1 month, and no significant difference at either time point with CASA ($P = 0.09$).

### Time to progression (TP) at 7 months

NTx was the only bone resorption marker able to discriminate reliably after 1 and 4 months of treatment between patients progressing within 7 months of commencing treatment (TP ≤ 7) and those with TP of > 7 months (Figure 2A). There was no significant differences in uCa and hyp levels between these groups. Both levels of CA15-3 and CASA were also significantly different in the two groups (Figure 2B, C).

### Predictive values

The DE of tAP was greatest at a 10% change, and for CA15-3 and CASA with a 10–20% change. To predict response, the DE of NTx and uCa was greatest with a 30% change, while to predict progression the DE with these markers was greatest with a 30–50% change (Figure 3). When some of these cut-offs values were used to build an algorithm, the best combination to predict response was NTx, tAP and clinical (pain score) response, although other combinations and even individual variables had similar results (Table 5). We also calculated the predictive values of an increase in markers of 20%, 30% and 50% whenever it occurred to predict for progression. The greatest DE of the resorption markers and CA15-3 was obtained with an increase of 50%, as shown in Table 6.

### Pain score

Five patients were pain-free without any analgesics at baseline and remained pain-free during the trial. These patients could not be included in this analysis. Four of them were in the PR/NC, and one in the PD, group. We correlated changes in the pain score with radiological changes at 4 months. PD patients had a significantly worse pain score when compared to PR/NC patients at 1 and 4 months. Five patients were considered as pain responders at 4 months. Four of them were in the PR/NC group. Four other patients were considered pain responders at 7 months, two were PR/NC and two were in the PD group.

Bone resorption and tumour marker levels were compared in clinical responders and non-responders. Only NTx showed a significant difference in mean values between the two clinical response groups; this was most marked after 4 months of treatment ($P \leq 0.01$) (Figure 4).

 *© Cancer Research Campaign 1999*

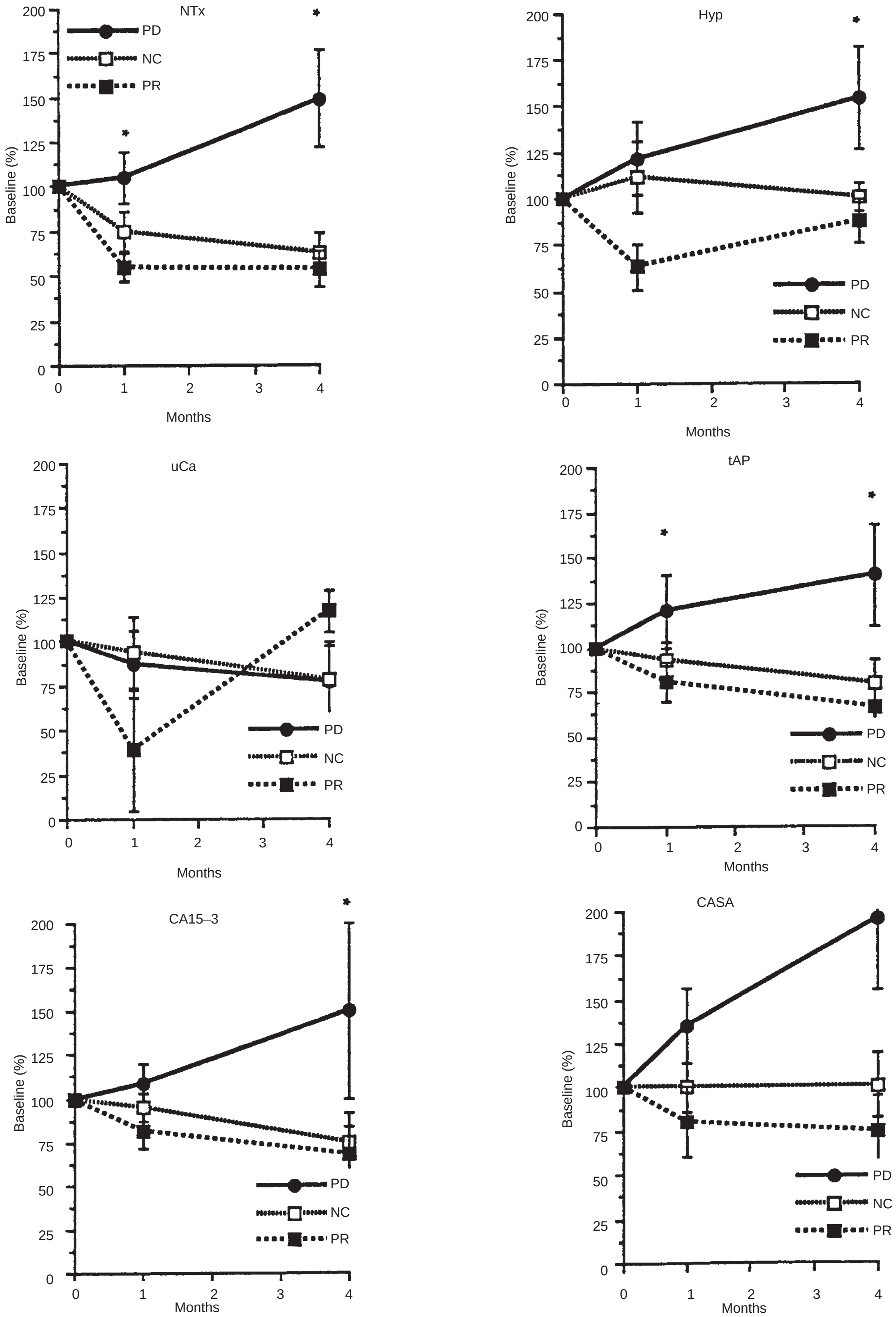

**Figure 1** Changes from baseline in bone and tumour markers by response category assigned at assessment of response after 4 months treatment. PR, *n* = 8; NC, *n* = 12; PD, *n* = 17. Significance levels between groups are shown as *$P \leq 0.05$, **$P < 0.01$. NTx = N-telopeptide; Hyp = hydroxyproline; uCa = urinary calcium; tAP = total alkaline phosphatase; CASA = cancer-associated serum antigen

© Cancer Research Campaign 1999

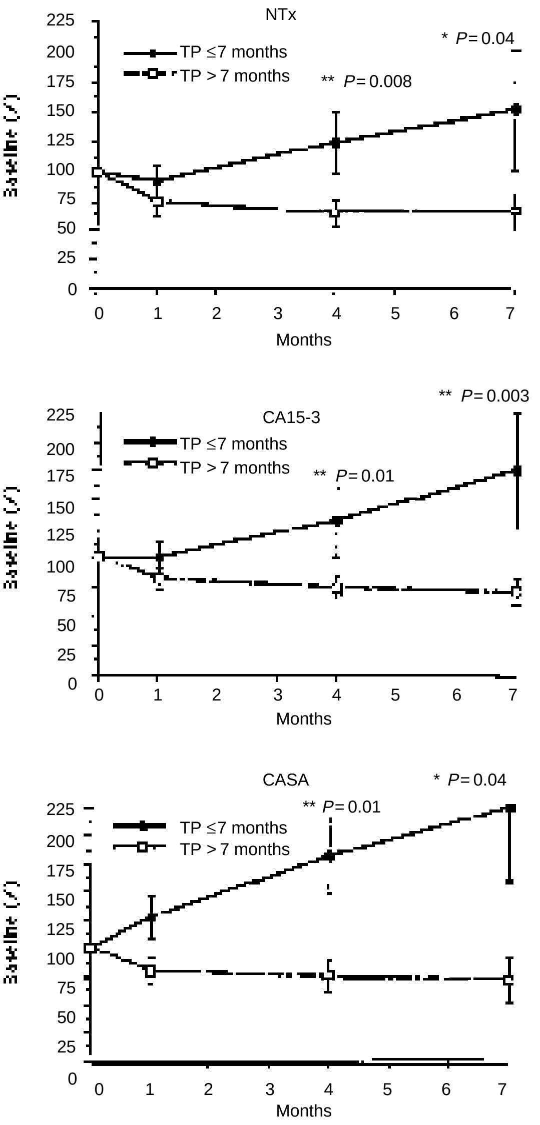

**Figure 2** Changes from baseline in NTx (top), CA15-3 (middle) and CASA (bottom) according to time to progression (TP) of < 7 months or ≥ 7 months. TP of < 7 months, *n* = 20; TP of ≥ 7 months, *n* = 13. Significance levels between groups are shown as *$P \leq 0.05$, ** $P < 0.01$

### Influence of underlying treatment

The choice of systemic anticancer treatment had no significant effect on the changes seen in bone resorption markers including NTx, but the changes seen in tumour markers were larger after chemotherapy than endocrine therapy. Patients receiving oral pamidronate had a significant reduction in NTx from baseline ($P < 0.001$) compared with no overall change in NTx in the placebo group indicating that, despite the rather poor tolerability and low absorption of this bisphosphonate, sufficient quantities were reaching the bone surface to influence bone metabolism.

### DISCUSSION

Bone is the only site of metastatic disease that has separate criteria for evaluation of response to treatment, based on bone repair and destruction rather than changes in tumour volume (Hayward et al, 1977). Assessing response to treatment in bone is more difficult than evaluation of disease in viscera and soft tissues where direct tumour measurements can usually be taken.

Because bone metastases inevitably perturb the function of normal bone cells, biochemical markers of bone resorption have been studied as possible alternative response criteria. We have shown that changes in NTx after 1–4 months of either endocrine treatment or chemotherapy reflect with reasonable accuracy the disease process as indicated by subsequent changes in plain radiographs. NTx was the only bone resorption marker either to correlate with radiological response at 4 and 7 months or to show a difference between pain responders and non-responders. NTx was also the bone resorption marker with the highest diagnostic efficiency to detect PD whenever it occurred; PD patients showed progressively higher values of NTx.

From our results we have proposed an algorithm based on the changes in NTx, CA15-3 and clinical response seen after only 1 month of treatment in order to predict eventual radiographic (objective) response. The algorithm was able to predict radiological response and time to progression correctly in 70% of patients. Prospective validation in a larger study should be attempted.

The changes seen in this study in collagen cross-link excretion are similar to those of a preliminary report on the effects of endocrine therapy (tamoxifen or megestrol acetate) in patients with bone metastases from breast cancer on the urinary excretion of the collagen cross-link deoxypyridioline (Dpd) measured by high performance liquid chromatography (Downey et al, 1994). After treatment, cross-links increased in patients with PD ($P < 0.05$), but remained unchanged or decreased in patients with stable disease or partial response. In another study, Blomqvist et al (1996) monitored the serum C-telopeptide of type 1 collagen (ICTP) and observed increasing levels of ICTP in PD patients, although a few patients with increasing ICTP subsequently showed a PR/NC.

None of the bone resorption or tumour markers could distinguish between NC and PR patients. In a recent trial in breast cancer, ICTP could also not distinguish between these two categories of response (Blomqvist et al, 1996). However, although the radiographic changes may differ in these two response categories, several studies have shown that the survival prospects in patients showing either stable disease for > 6 months or a partial response are very similar (Coleman and Rubens 1985, Howell et al, 1988). Our data support the notion that, in fact, these categories are similar in terms of both biochemical response and clinical outcome.

There is only one preliminary report on the use of collagen cross-link measurements following systemic therapy associated with bisphosphonates (Lipton et al, 1995). In this trial, 51 patients (49 breast cancer, two myeloma) with bone metastases were treated with either pamidronate 90 mg intravenously monthly or placebo in addition to standard systemic therapy. During the 6 months of the trial, NTx was the marker that showed the most significant decrease ($P = 0.0006$), while there was a smaller difference with deoxypyridinoline ($P = 0.03$) and no detectable difference with pyridinoline.

UCa excretion was low at baseline, showed no correlation with radiological response at 4 months or 7 months, and did not strongly correlate with either NTx at baseline, or with hyp or NTx after treatment. In addition, uCa was the resorption marker with the least diagnostic efficiency to detect PD. This contradicts the

*© Cancer Research Campaign 1999*

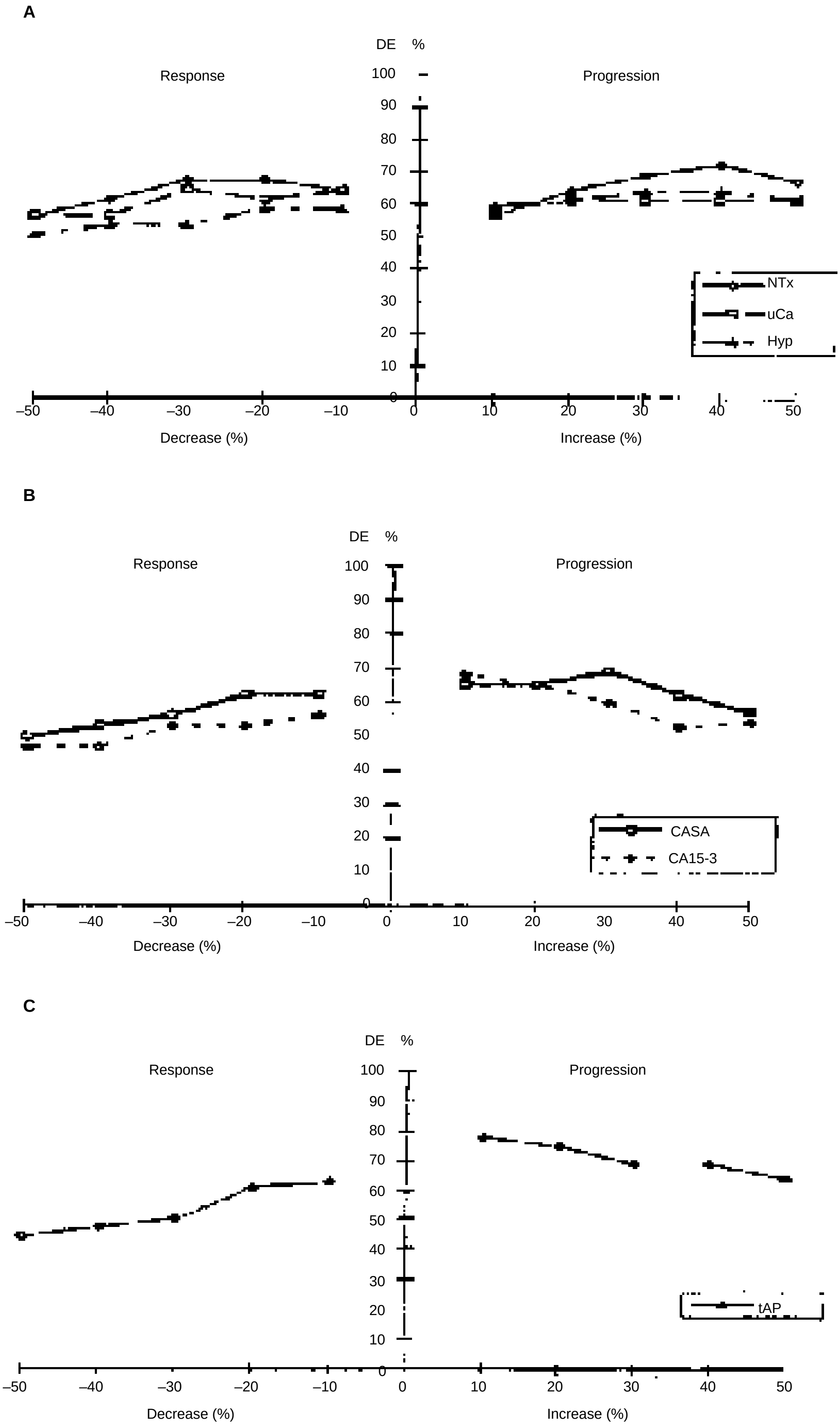

**Figure 3** Diagnostic efficiency (DE) of changes at 1 month from baseline in bone resorption markers (**A**), tumour markers (**B**) and tAP (**C**) to predict objective response at 4 months

*© Cancer Research Campaign 1999*

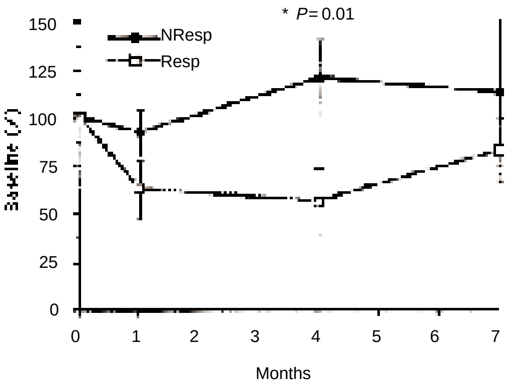

**Figure 4** Changes in NTx in pain responders (Resp, *n* = 9) and non-responders (NResp, *n* = 21)

value of changes in uCa excretion reported in two previous studies where there appeared to be a good correlation between response to treatment and changes in uCa excretion (Campbell et al, 1987; Coleman et al, 1988). However, both of these trials evaluated patients with established progressive bone metastases in the absence of bisphosphonates. In this study of patients with newly diagnosed bone metastases, the disturbance in bone resorption was less profound and half of the patients were receiving bisphosphonates which, through the stimulation of parathyroid hormone production and consequent stimulation of renal tubular reabsorption of calcium, are known to suppress calcium excretion independent of any skeletal effects of the treatment (Vinholes et al, 1997*a*).

Of the two tumour markers assessed, only CA15-3 showed a correlation with radiological response at 4 months. There was no correlation between tumour and bone resorption markers suggesting they are reflecting different aspects of the disease process and are probably complementary. The effects of chemotherapy on tumour markers were more pronounced than the effects of endocrine treatment, possibly suggesting greater anti-tumour efficacy of chemotherapy in this sample of patients.

In conclusion, CA15-3 is the most sensitive marker to detect newly diagnosed bone metastases and NTx the most useful resorption marker to assess response to treatment. Rising levels of NTx and tumour markers generally indicate progression. NTx is a sensitive resorption marker to detect the biochemical effects of oral pamidronate. NTx was the only marker that correlated with subjective response. We hope to test the algorithm to predict response in bone to treatment and time to progression in a large prospective study.

### REFERENCES

Blomqvist C, Sarna S, Elomaa I et al (1996) Markers of type I collagen degradation and synthesis in the monitoring of treatment response in bone metastases from breast carcinoma. *Br J Cancer* **73**: 1074–1079

Blumsohn A, Naylor RA, Eastell R et al (1994) The effects of calcium supplementation on the circadian rhythm of bone resorption. *J Clin Endoc Metab* **79**: 730–735

Bonde M, Qvist P, Christiansen C et al (1995) Applications of an enzyme immunoassay for a new marker of bone resorption (Crosslaps): follow-up on hormone replacement therapy and osteoporosis risk assessment. *J Clin Endocrinol Metab* **80**: 864–868

Campbell FC, Blamey RW, Woolfson AMJ, Elston CW and Hosking DT (1983) Calcium excretion CaE in metastatic breast cancer. *Br J Surg* **70**: 202–204

Coleman RE (1994) Evaluation of bone disease in breast cancer. *Breast* **3**: 73–78

Coleman RE (1999) Monitoring of bone metastases. *Eur J Cancer* **34**: 252–259

Coleman RE and Rubens RD (1985) Bone metastases and breast cancer. *Cancer Treat Rev* **12**: 251–270

Coleman RE, Whitaker KB, Moss DW, Mashiter G, Fogelman I and Rubens RD (1988) Biochemical prediction of response of bone metastases to treatment. *Br J Cancer* **58**: 205–210

Downey S, Eastell R, Howell A et al (1994) Pyridinoline excretion can monitor bone metastases in breast cancer. *Breast Cancer Res Treat* **32**: 83

Galasko CSB (1994) Diagnosis of skeletal metastases and assessment of response to treatment. In *Metastatic Bone Disease*, Diel IJ, Kaufmann M and Bastert G (eds), pp. 93–107. Springer Verlag: Heidelberg.

Hanson D, Weis MAE, Bollen AM et al (1992) A specific immunoassay for monitoring human bone resorption: quantification of type 1 collagen cross-linked N-telopeptides in urine. *J Bone Miner Res* **7**: 1251–1258

Hayward JL, Carbone PP, Heuson JC, Kumaoka S, Segaloff A and Rubens RD (1977) Assessment of response to therapy in advanced breast cancer. *Cancer* **3**: 1389–1394

Howell A, Mackintosh J, Jones M, Radford J, Wagstaff J and Selwood RA (1988) The definition of the ‘no change’ category in patients treated with endocrine therapy and chemotherapy for 6 advanced carcinoma of the breast. *Eur J Cancer* **24**: 1567–1572

Lipton A, Hortobagyi G, Reitsma D et al (1995) Markers of bone resorption in patients treated with pamidronate. *Bone* **17**: 615

Robertson JFR, Pearson D, Price MR, Selby C, Blamey R and Howell A (1991) Objective assessment of therapeutic response in breast cancer using tumour markers. *Br J Cancer* **64**: 757–763

Vinholes JJ, Guo C-Y, Purohit OP, Eastell R and Coleman RE (1996) Metabolic effects of pamidronate in patients with metastatic bone disease. *Br J Cancer* **73**: 1089–1095

Vinholes J, Guo C-Y, Purohit OP, Eastell R and Coleman RE (1997*a*) Evaluation of new bone resorption markers in a randomized comparison of pamidronate or clodronate for hypercalcaemia of malignancy. *J Clin Oncol* **15**: 131–138

Vinholes J, Purohit OP, Abbey ME, Eastell R and Coleman R (1997*b*) Relationships between biochemical and symptomatic response in a double-blind randomised trial of pamidronate for metastatic bone disease. *Ann Oncol* **8**: 1243–1250

*© Cancer Research Campaign 1999*
